# SYCP1 head-to-head assembly is required for chromosome synapsis in mouse meiosis

**DOI:** 10.1126/sciadv.adi1562

**Published:** 2023-10-20

**Authors:** Katherine Kretovich Billmyre, Emily A. Kesler, Dai Tsuchiya, Timothy J. Corbin, Kyle Weaver, Andrea Moran, Zulin Yu, Lane Adams, Kym Delventhal, Michael Durnin, Owen Richard Davies, R. Scott Hawley

**Affiliations:** ^1^ Stowers Institute for Medical Research, Kansas City, MO 64110, USA.; ^2^ Wellcome Centre for Cell Biology, Institute of Cell Biology, University of Edinburgh, Michael Swann Building, Max Born Crescent, Edinburgh EH9 3BF, UK.; ^3^ Department of Molecular and Integrative Physiology, University of Kansas Medical Center, Kansas City, KS 66160, USA.

## Abstract

In almost all sexually reproducing organisms, meiotic recombination and cell division require the synapsis of homologous chromosomes by a large proteinaceous structure, the synaptonemal complex (SC). While the SC’s overall structure is highly conserved across eukaryotes, its constituent proteins diverge between phyla. Transverse filament protein, SYCP1, spans the width of the SC and undergoes amino-terminal head-to-head self-assembly in vitro through a motif that is unusually highly conserved across kingdoms of life. Here, we report creation of mouse mutants, *Sycp1^L102E^* and *Sycp1^L106E^*, that target SYCP1’s head-to-head interface. L106E resulted in a complete loss of synapsis, while L102E had no apparent effect on synapsis, in agreement with their differential effects on the SYCP1 head-to-head interface in molecular dynamics simulations. In *Sycp1^L106E^* mice, homologs aligned and recruited low levels of mutant SYCP1 and other SC proteins, but the absence of synapsis led to failure of crossover formation and meiotic arrest. We conclude that SYCP1’s conserved head-to-head interface is essential for meiotic chromosome synapsis in vivo.

## Teaser

Head-to-head assembly of SYCP1 is necessary for meiotic chromosome synapsis and fertility in mice.

## INTRODUCTION

Meiotic cell division is a conserved process that is necessary for sexual reproduction via the creation of haploid gametes (e.g., eggs and sperm). Errors in meiosis can lead to aneuploid gametes containing the incorrect number of chromosomes (too many or too few). Aneuploidy in gametes is a leading cause of infertility and birth defects in humans (*1*). Ten to 15% of couples deal with infertility, and 40 to 50% of those cases are due to male infertility (*2*, *3*). Of those male cases, about 10% are estimated to be due to nonobstructive azoospermia where no sperm are produced (*2*, *4*).

The early events necessary for correct chromosome segregation are the formation of double-strand breaks (DSBs), homologous pairing, synapsis, and the repair of DSBs into crossovers or noncrossovers (*5*, *6*). During murine meiosis, the first step is the induction of ~400 DSBs, which leads to homology searches between homologs that establish pairing (*7*). Next, the synaptonemal complex (SC), a large multiprotein structure, assembles between homologs. Full assembly of the SC is necessary for DSBs to be repaired into crossovers (*5*, *8*). In mammalian models of meiosis, meiotic arrest due to errors in early meiosis results in a complete loss of gamete production, termed nonobstructive azoospermia in males and ovarian insufficiency in females (*2*, *9*, *10*).

**Fig. 1.**
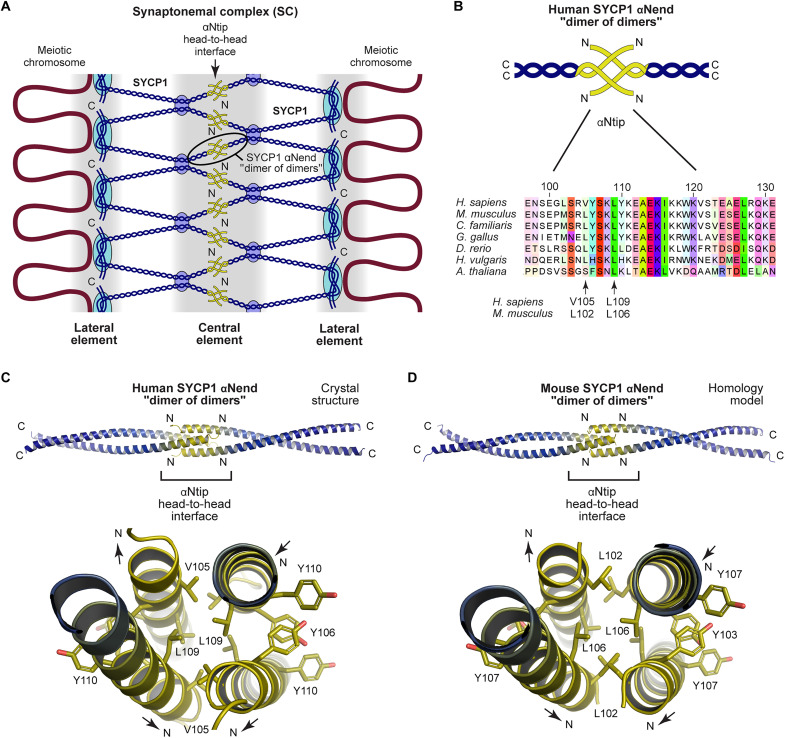
Mammalian SYCP1 self-assembly is mediated by αNtip head-to-head interactions. (**A**) Current model of SYCP1 structure within the SC (*23*, *24*). N-terminal self-assembly of SYCP1 is mediated by the αNend of the α-helical core (amino acids 101 to 175 in humans), which undergoes head-to-head dimer of dimers interaction through its αNtip (amino acids 101 to 111 in humans). Dark blue circles represent the tetramer interface between parallel dimers. Light blue circles mark anchoring of SYCP1 to the chromosome axes through back-to-back assembly of C termini. (**B**) Schematic of the SYCP1 αNend head-to-head structure (top) and multiple sequence alignment of SYCP1 surrounding its conserved αNtip (bottom). Amino acids are colored by chemical properties and according to conservation (*46*). (**C**) Crystal structure of human SYCP1 αNend, highlighting the roles of amino acids V105 and L109 within the hydrophobic core of the αNtip head-to-head interface. (**D**) Homology model of mouse SYCP1 αNend showing the predicted positions of amino acids L102 and L106 (which are equivalent to human amino acids V105 and L109, respectively) at the αNtip head-to-head interface.

The SC is a highly conserved structure and is present in almost all eukaryotes that reproduce sexually (*11*–*13*). The structure is tripartite with lateral elements interacting with the DNA on the exterior of the structure and a central region that spans the width between the homologs (*14*). The key proteins that make up the mammalian SC are lateral element proteins (SYCP2 and SYCP3) (*15*, *16*), a transverse filament protein (SYCP1) (*8*, *17*), and multiple central element (CE) proteins (SYCE1, SIX6OS1, SYCE2, SYCE3, and TEX12) (*18*–*22*). Extensive work has shed light on the molecular structures of SC proteins and how SC proteins interact with each other to fully synapse chromosomes. SYCP1 contains a large coiled-coil domain flanked by unstructured regions. The coiled-coil is organized with its N and C termini within the central region and lateral elements, respectively, and undergoes several self-assembly interactions that are thought to facilitate formation of a lattice-like SC structure (*23*, *24*). In the midline, SYCP1 dimers interact laterally to form tetramers and opposing dimers interact head to head via the N-terminal ends of the coiled-coil structure, mediated by short motifs at their N-terminal tips (Fig. 1A) (*23*). In the lateral element, the C-terminal end of the coiled-coil self-associates and binds to DNA along with the unstructured C terminus of SYCP1 (*23*). The SYCP1 lattice is supported by the presence of CE proteins that are involved in synaptic initiation, remodeling, and elongation. SYCE3 interacts directly with SYCP1 and integrates into the SYCP1 lattice such that its interactions with SYCE1/SIX6OS and SYCE2/TEX12 recruit them to the SC (*24*). If SYCE3 cannot be recruited, then the rest of the CE proteins are lost from the homologs (*19*). SYCE1 and SIX6OS are thought to be initiation factors that function as structural supports between SYCP1 proteins (*25*, *26*). TEX12 and SYCE2 are potentially elongation factors, as they form a complex that self-assembles into long fibers that may help to stabilize the SYCP1 lattice as it extends along the chromosomes (*27*). Null mutants for any of the CE proteins exhibit failed synapsis and are male and female sterile (*8*, *18*, *19*, *21*, *22*). Understanding how all the SC proteins interact with each other is necessary to establish how the tripartite SC assembles and how the SC functions during meiosis.

The overall tripartite structure of the SC is highly conserved between organisms, yet there is very little conservation at the amino acid sequence level (*11*, *28*). Nevertheless, SYCP1 contains two regions that are highly conserved throughout vertebrates, which correspond to the self-assembly sites at the N- and C-terminal ends of the coiled-coil domain (*29*, *30*). The N-terminal motif can also be identified within *Arabidopsis thaliana* ZYP1, indicating conservation that spans two kingdoms of life but is not found in the common meiosis model organisms *Saccharomyces cerevisiae*, *Caenorhabditis elegans*, and *Drosophila melanogaster*. This motif corresponds to the αNend coiled-coil, including the αNtip sequence that mediates head-to-head interaction of human SYCP1 molecules, which can be disrupted by introduction of mutation V105E/L109E in vitro (*23*). Accordingly, αNtip-mediated head-to-head assembly of transverse filament proteins is predicted to constitute an essential and evolutionarily conserved mechanism in SC assembly. However, this has not formally been tested in vivo.

Here, we used CRISPR-Cas9 to disrupt αNtip-mediated head-to-head assembly of SYCP1 by replacing two leucine amino acids within the mouse sequence, L102 and L106 (corresponding to human amino acids V105 and L109), with glutamic acids. We analyzed *Sycp1^L102E^*, *Sycp1^L106E^*, and the double mutant *Sycp1*^*L102E*/*L106E*^ for their effects on homologous chromosome pairing, SC formation, crossover formation, and fertility. In agreement with molecular dynamics (MD) simulations, *Sycp1^L102E^* mice had no apparent SC defects, but synapsis was defective in both *Sycp1^L106E^* and *Sycp1*^*L102E*/*L106E*^ mice. Nuclei were arrested in a zygotene-like/pachytene-like state where homologs were aligned but not synapsed. SYCP1 staining was present on homologs but at reduced levels. In addition, male mice were infertile with small testes likely due to meiotic arrest. Thus, we uncover that a single conserved leucine (m.L106/h.109) in SYCP1 is necessary for SC assembly. Therefore, αNtip-mediated head-to-head assembly of SYCP1 is essential for meiotic chromosome synapsis in vivo.

## RESULTS

### SYCP1 head-to-head self-assembly depends on conserved residue h.L109/m.L106

The overall structure and function of the SC are highly conserved across sexually reproducing organisms. However, the amino acid sequence of SC proteins and even the number of SC proteins can vary widely between organisms. The most highly conserved regions of SYCP1 correspond to sequences responsible for its self-assembly. In the midline, the N-terminal end of the coiled-coil (αNend; amino acids h.101 to h.175) undergoes head-to-head self-assembly in vitro through dimer-of-dimer interactions that are mediated by their αNtips (amino acids h.101 to h.111) (Fig. 1A). Sequence conservation is particularly strong in this region of the protein, extending as far as *A. thaliana* (Fig. 1B). We previously demonstrated that head-to-head assembly of human SYCP1 in vitro could be blocked by deletion of its αNtip or mutation of two conserved αNtip amino acids V105E and L109E (*23*). These mutations target residues that are buried in the hydrophobic core of the head-to-head interface, disrupting the structure through introduction of negative charge.

While human SYCP1 amino acids V105 and L109 are conserved as leucines in many vertebrate sequences, we noticed that only L109 is conserved in *A. thaliana* (Fig. 1B). In the crystal structure of human SYCP1 αNend, L109 side chains are buried at the center of the structure, whereas V105 residues are closer to the surface (Fig. 1C) (*23*). The same features are observed in a homology model of mouse SYCP1 αNend, in which L106 (equivalent of h.L109) side chains are completely buried, whereas L102 (equivalent of h.V105) residues are closer to the molecular surface (Fig. 1D). These findings suggested that m.L106/h.L109 may have an essential conserved role in αNtip head-to-head assembly of SYCP1, whereas the role of m.L102/h.V105 may be more peripheral.

**Fig. 2.**
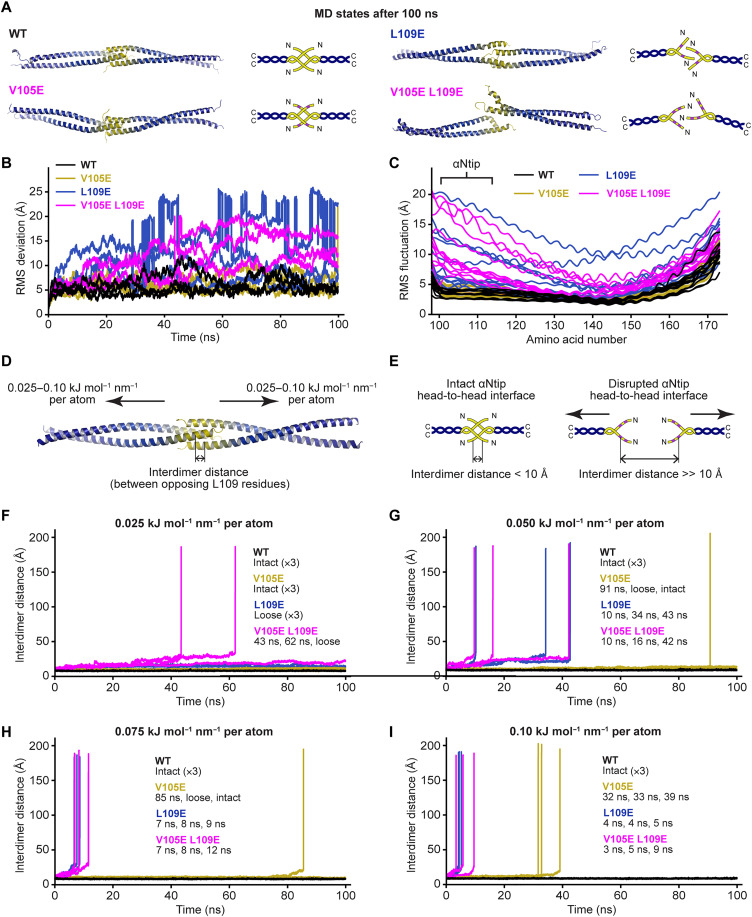
MD simulations of the human SYCP1 αNend dimer of dimers structure. (**A** to **C**) MD simulations of human SYCP1 αNend WT, V105E, L109E, and V105E/L109E over 100-ns trajectories, in explicit solvent at 37°C (*n* = 3). (A) Representative states after 100-ns simulations. (B) Overall RMS deviations for all replicates. (C) Individual amino acid RMS fluctuations, shown for one chain of each replicate, and indicating the position of the αNtip sequence. (**D** to **I**) Steered MD simulations in which forces of between 0.025 and 0.10 kJ mol^−1^ nm^−1^ were applied to each atom, directed along the axial axis, in opposite directions for the two constituent dimers. (D) Disruption of the αNtip head-to-head interface was assessed by measuring the distance between L109 amino acids of opposing dimers. (E) Interdimer distances were less than 10 Å when the interface remained intact and increased substantially when dimers drifted apart following interface disruption. (F to I) Interdimer distances plotted for each replicate of SYCP1 αNend WT, V105E, L109E and V105E/L109E, when under opposing forces of (F) 0.025, (G) 0.050, (H) 0.075, and (I) 0.10 kJ mol^−1^ nm^−1^ per atom. Interface disruption is marked by a sudden increase in interdimer distance, and times of disruption are indicated for all replicates.

We tested the roles of human SYCP1 amino acids V105 and L109 in silico by performing MD simulations of the αNend “dimer of dimers” structure using wild-type (WT), V105E, L109E, and V105E/L109E sequences (amino acids 101 to 175). Simulations were run for 100 ns, in explicit solvent, at 37°C, in triplicate. For the WT and V105E structures, the head-to-head interface remained essentially intact (Fig. 2A and fig. S1), with overall root mean square (RMS) deviations of around 5 Å (Fig. 2B and fig. S1), which were mostly attributed to large fluctuations within the C-terminal ends of the coiled-coils (Fig. 2C). In contrast, L109E and V105E/L109E structures underwent substantial distortions in which the head-to-head interface opened up into a loose structure, and in some cases, the two interacting dimers dissociated (Fig. 2A and fig. S1). This was apparent in the high overall RMS deviations of up to 20 Å (Fig. 2B and fig. S1) and large RMS fluctuations of the αNtip head-to-head assembly residues.

We next performed steered MD simulations by applying axial forces in opposite directions for the two constituent dimers of the αNend structure, such that the head-to-head interacting dimers were pulled apart (Fig. 2D). In this setup, opposing dimers would drift apart upon disruption of the head-to-head interface. This provided a simple means for determining the point of dissociation by recording the distance between L109 residues of opposing dimers (interdimer distance); the distance remained small (less than 10 Å) for intact interfaces and increased to large distances (much greater than 10 Å) upon disruption of the head-to-head interface (Fig. 2E). We performed 100-ns simulations for WT, V105E, L109E, and V105E/L109E sequences, with forces between 0.025 and 0.10 kJ mol^−1^ nm^−1^ per atom (corresponding to total forces per dimer of approximately 100 to 400 pN), in explicit solvent, at 37°C, in triplicate (Fig. 2, F to I). The use of axial forces, in opposing directions on bioriented dimers, bears similarity to tension forces attempting to pull apart the midline synapsis between meiotic chromosomes. However, we do not know the magnitude and nature of tension forces acting upon synapsed chromosomes during the stages of meiosis in vivo. Hence, any similarity is qualitative rather than quantitative. The range of forces was determined empirically from preliminary studies in which we established the minimum and maximum forces in which mutant structures and WT structures were consistently disrupted or retained, respectively, over the timeframe of 100-ns simulations. In all conditions, the WT structure remained intact throughout (Fig. 2, F to I). The V105E structure remained intact, albeit with a loose/distorted interface at forces up to 0.075 kJ mol^−1^ nm^−1^ but underwent dissociation during simulations using forces of 0.10 kJ mol^−1^ nm^−1^ (Fig. 2, F to I). In contrast, L109E and V105E/L109E structures readily dissociated at similar time points at forces between 0.050 and 0.10 kJ mol^−1^ nm^−1^ (Fig. 2, G to I). There was a slight difference at the lowest force of 0.025 kJ mol^−1^ nm^−1^, in which the L109E structure only loosened/distorted but did not dissociate, whereas the V105E/L109E structure dissociated in two out of three replicates (Fig. 2F).

To confirm our findings, we performed MD simulations using the mouse SYCP1 αNend modeled structure. In 100-ns unsteered simulations, the WT, L102E, and L106E structures remained largely intact, with overall RMS deviations of 5 to 7 Å (fig. S2A). In contrast, the L102E/L106E structure opened up into a distorted loose structure, with RMS deviations of approximately 15 Å (fig. S2A). In steered MD simulations, imposing axial forces of 0.050 kJ mol^−1^ nm^−1^ per atom, the WT and L102E structures remained largely intact, whereas L106E and L102E/L106E structures dissociated at 23 to 54 ns and 6 to 15 ns, respectively (fig. S2B). Hence, these simulations confirm that the head-to-head interface of the mouse structure is destabilized by L106E and L102E/L106E mutations.

In summary, our MD simulations indicate that h.L109E/m.L106E is almost as effective as the V105E/L109E double mutation in disrupting αNtip-mediated head-to-head assembly of SYCP1. Nevertheless, there was a slightly increased destabilization of the double-mutant structure, and the individual V105E structure was slightly less stable than the WT structure. These findings are explained by the structure in which the peripheral location of the h.V105E/L109E (m.L102E/L106E) amino acids likely allows the glutamate mutants to orient with the negative charge exposed to solvent, which is not possible for the fully buried h.L109 amino acids (Fig. 1C). Moreover, our in silico analysis suggests that the h.L109E/m.L106E mutation, but not the h.V105E/m.L102E mutation, is sufficient to disrupt αNtip-mediated SYCP1 head-to-head assembly.

### *Sycp1^L106E^* and *Sycp1^L102E/L106E^* homozygous mice exhibit infertility and spermatocyte death

**Fig. 3.**
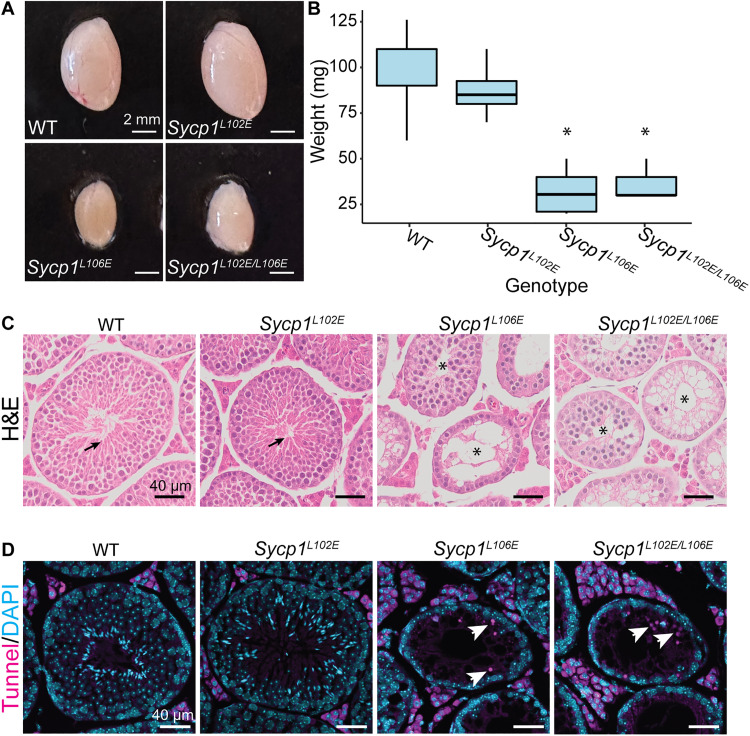
*Sycp1^L106E^* and *Sycp1^L102E/L106E^* mice have smaller testes and exhibit increased apoptosis. (**A**) Images of whole testes dissected from 9-week-old mice. (**B**) *Sycp1^L106E^* and *Sycp1^L102E/L106E^* testes are reduced in weight compared to control and *Sycp1^L102E^* mice. *N* values WT = 8, *Sycp1^L102E^* = 6 (*P* = 0.0800), *Sycp1^L106E^* = 4 (*P* < 0.0001), *Sycp1^L102E/L106E^* = 5 (*P* < 0.0001). *P < 0.0001. (**C**) Hematoxylin and eosin (H&E) staining of paraffin sections showing normal seminiferous tubules in WT and *Sycp1^L102E^* testes with mature sperm (black arrow) but smaller empty seminiferous tubules in *Sycp1^L106E^* and *Sycp1^L102E/L106E^* testes (marked with an asterisk). (**D**) Sections stained with TUNEL for apoptotic cells (magenta) and DAPI (cyan) marking DNA. Apoptotic cells marked with white arrows. Scale bars, 2 mm (A) and 40 μm (C and D). Minimum of three animals analyzed per a genotype.

To test the role of αNtip-mediated SYCP1 head-to-head assembly in vivo, we used CRISPR-Cas9 to create mice harboring αNtip L102E and L106E mutations that correspond to the human V105E and L109E mutations described above (see Materials and Methods and fig. S3). We first tested the fertility of male mice carrying *Sycp1^L102E^*, *Sycp1^L106E^*, or *Sycp1*^*L102E*/*L106E*^ mutations. Fertility assays between homozygous mutant males and C57BL/6J females confirmed that homozygous *Sycp1^L102E^* male mice were fertile and *Sycp1^L106E^* and *Sycp1*^*L102E*/*L106E*^ male mice were sterile (table S1). Furthermore, testes from *Sycp1^L106E^* and *Sycp1*^*L102E*/*L106E*^ mice were 32 and 35% the weight of WT testes at 10 weeks, respectively (Fig. 3, A and B). The reduction in testes size in *Sycp1^L106E^* and *Sycp1^L102E/L106E^* mice was due to atrophied seminiferous tubules that did not contain any postmeiotic spermatids (Fig. 3C). In addition, we found that *Sycp1^L102E^* females were fertile and *Sycp1^L106E^* and *Sycp1*^*L102E*/*L106E*^ females were sterile when mated with C57BL/6J males (table S1). However, we did not study female meiosis any further. This phenotype is similar to the arrest seen in *Sycp1^−/−^* null mice and other SC mutants (*8*, *19*, *21*, *22*, *25*, *31*). To further investigate the cause of the reduced testes size, terminal deoxynucleotidyl transferase–mediated deoxyuridine triphosphate nick end labeling (TUNEL) staining was performed on testes sections to visualize apoptotic cells to confirm meiotic arrest. In sections of *Sycp1^L106E^* and *Sycp1*^*L102E*/*L106E*^ testes, the percentage of seminiferous tubules exhibiting five or more apoptotic nuclei increased compared to *Sycp1^L102E^* or WT mice (Fig. 3D and table S2; WT: 1.5%, *Sycp1^L102E^*: 2.2%, *Sycp1^L106E^*: 16.1%, and *Sycp1*^*L102E*/*L106E*^: 11.8%). This level of apoptosis mirrors that seen in other SC mutants where male meiosis is prematurely arrested leading to cell death, decreased seminiferous tubule size, and no mature spermatids (*8*, *19*, *21*, *22*, *25*, *31*).

### SC assembly in *Sycp1^L106E^* and *Sycp1^L102E/L106E^* homozygous males is defective

**Fig. 4.**
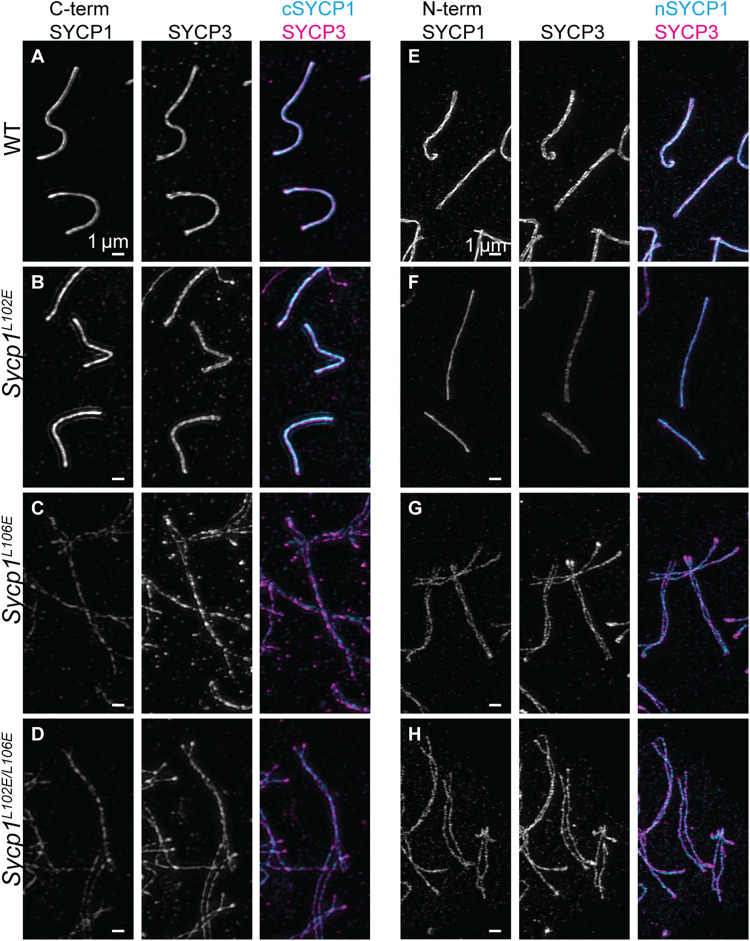
Mutant SYCP1 is present on the chromosome axes in pachytene-like nuclei in *Sycp1^L106E^* and *Sycp1^L102E/L106E^* mutants. Pachytene/pachytene-like chromosomes stained with antibodies against C-terminal SYCP1 (**A** to **D**) or N-terminal SYCP1 (**E** and **F**) and SYCP3 were imaged using structured illumination microscopy (SIM) in (A) and (E) WT, (B) and (F) *Sycp1^L102E^*, (C) and (**G**) *Sycp1^L106E^*, and (D) and (**H**) *Sycp1^L102E/L106E^* mice. The first panel is SYCP1 (gray), the second is SYCP3 (gray), and the third is a merge of SYCP1 (cyan) and SYCP3 (magenta) staining. All images are at the same exposure. Scale bars, 1 μm. Minimum of three animals analyzed per a genotype.

To determine whether the meiotic arrest seen in *Sycp1^L106E^* and *Sycp1*^*L102E*/*L106E*^ males was due to an underlying SC defect, SC structure was examined using superresolution microscopy on chromosome spreads stained with antibodies against SYCP1(C terminus) and SYCP3. WT and *Sycp1^L102E^* nuclei showed normal progression of SC assembly (Fig. 4, A and B, and fig. S4). SYCP3 localized to the axis before SYCP1 in late leptotene and SYCP3 and SYCP1 colocalized showing fully aligned and synapsed chromosomes in pachytene (Fig. 4, A and B, and fig. S4). However, in *Sycp1^L106E^* and *Sycp1*^*L102E*/*L106E*^ mice, the chromosomes aligned, but the SYCP1 and SYCP3 signals appeared further apart than in controls making it difficult to properly stage meiotic nuclei. However, SYCP3 and SYCP1 staining colocalized along the chromosome axes in what appeared to be pachytene-like nuclei (Fig. 4, C and D, and fig. S4). To confirm that the chromosomes in *Sycp1^L106E^* and *Sycp1*^*L102E*/*L106E*^ mice were not synapsed, we measured the distance between the chromosome axes in superresolution images. In WT mice, the distance between synapsed chromosomes is on average 150 nm. In both WT and *Sycp1^L102E^* mice, the chromosomes had an average distance of 132 and 145 nm respectively (fig. S5; not significant). However, in *Sycp1^L106E^* and *Sycp1*^*L102E*/*L106E*^ mice, this distance increased significantly to a mean distance of 365 and 332 nm, respectively (fig. S5; *P* < 0.0001). These data support a full loss of synapsis in both *Sycp1^L106E^* and *Sycp1*^*L102E*/*L106E*^ mutants. Because SYCP1 was present along the entire chromosome in *Sycp1^L106E^* and *Sycp1*^*L102E*/*L106E*^ nuclei, it indicated that the nuclei had progressed past zygotene and therefore were arrested in more of a pachytene-like state, although the homologs were not synapsed. Last, sperm heads were never observed in *Sycp1^L106E^* and *Sycp1*^*L102E*/*L106E*^ males but were present in both WT and *Sycp1^L102E^* mice.

We observed that when the SYCP1 signal spanned the length of the chromosomes in *Sycp1^L106E^* and *Sycp1*^*L102E*/*L106E*^ pachytene-like nuclei, the signal of C-terminal SYCP1 was fainter than in WT nuclei (Fig. 4 and figs. S4 and S5; WT versus L106E: *P* = 0.00006, WT versus L102E/L106E: *P* < 0.00001). This result confirmed that these mutations did not result in a complete loss of SYCP1 protein. There was also a decrease in C-terminal SYCP1 staining in *Sycp1^L102E^* mutants compared to controls (fig. S5; WT versus L102E: *P* = 0.0006). However, SYCP1 intensity in *Sycp1^L102E^* mutants was still significantly greater than in *Sycp1^L106E^* and *Sycp1*^*L102E*/*L106E*^ nuclei (fig. S5; L102E versus L106E: *P* = 0.0005, L102E versus L102E/L106E: *P* = 0.0048). Together, this suggests that the L102E mutation is insufficient to disrupt synapsis but may affect SYCP1 localization or stability.

### *Sycp1^L106E^* and *Sycp1^L102E/L106E^* homozygotes fail to mature DSBs into crossovers

**Fig. 5.**
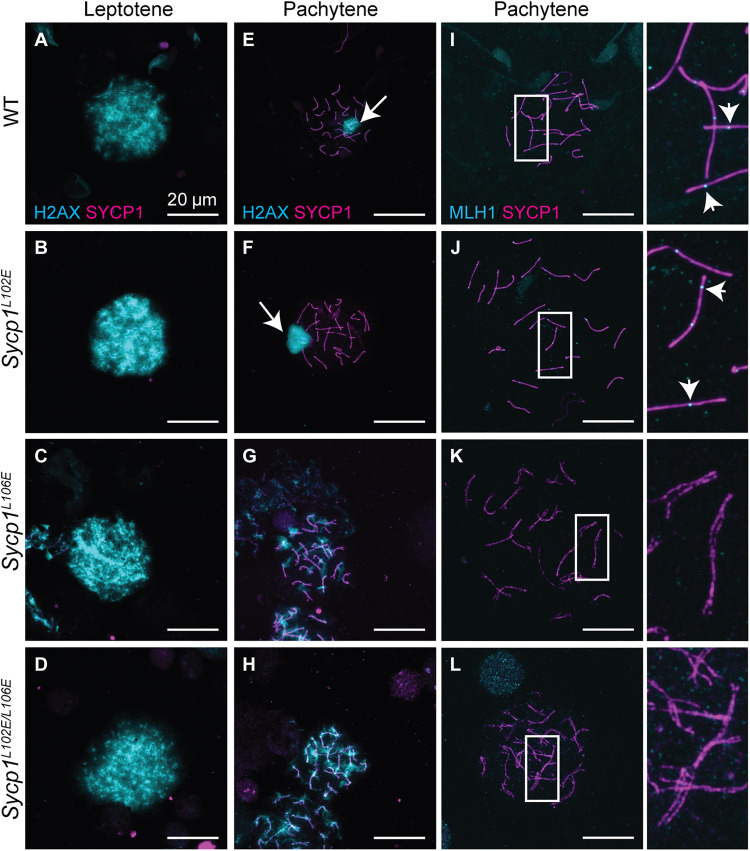
DSBs are not efficiently repaired into crossovers in *Sycp1^L106E^* and *Sycp1^L102E/L106E^* mutants. (**A** to **D**) Leptotene and (**E** to **H**) pachytene/pachytene-like nuclei stained with γH2AX marking DSBs (cyan) and SYCP1 (magenta). *XY* bodies are marked with white arrows. (**I** to **L**) Pachytene/pachytene-like nuclei stained with MLH1 marking class I crossovers (cyan) and SYCP1 (magenta). MLH foci are marked with white arrowheads. SYCP1 staining in *Sycp1^L106E^* and *Sycp1^L102E/L106E^* mutants has been brightened compared to controls to visualize the chromosomes easier. Scale bars, 20 μm, magnified panel is 3.2×. Minimum of three animals analyzed per a genotype.

During WT meiosis, DSBs are made before pairing and synapsis. After synapsis, they are repaired into crossovers and noncrossovers. However, in mutants that lack full synapsis, DSBs are never fully repaired, and crossovers do not occur (*5*). While each mutant form of SYCP1 was still localized to the axis in *Sycp1^L106E^* and *Sycp1*^*L102E*/*L106E*^ mice, homologous chromosomes never appeared to synapse (Fig. 4). γH2AX, an antibody that marks chromatin containing DNA damage, including SPO11-induced breaks (*32*), was used to examine DSB repair in WT and SYCP1 mutant mice. In leptotene, γH2AX staining was present in a cloud over all the chromosomes in WT and in all mutants (Fig. 5, A to D). In WT, γH2AX was restricted at pachytene to a haze surrounding the *XY* body, which is condensed silenced chromatin present on the *X* and *Y* chromosome in male meiosis (Fig. 5E). *Sycp1^L102E^* mice exhibited a similar pattern to WT controls in pachytene with γH2AX only present at the *XY* body and no staining on the rest of the chromosomes (Fig. 5F, WT: 35 of 35 nuclei, L102E: 40 of 40 nuclei showed only *XY* body staining). This contrasted with *Sycp1^L106E^* and *Sycp1*^*L102E*/*L106E*^ mice where γH2AX signal remained present on all the chromosomes, and there was no clear *XY* body (Fig. 5, G and H; L106E: 40 of 40 nuclei, L102E/L106E: 58 of 58 nuclei showed excessive γH2AX staining). The lack of *XY* body has also been reported in other mutants lacking synapsis where DSBs are not properly repaired, and the *X* and *Y* chromosomes do not synapsis properly (*22*, *24*–*26*, *31*).

In addition, we examined the samples for the presence of MLH1, a mismatch repair protein required for class I crossovers (*33*). At pachytene, both WT and *Sycp1^L102E^* mice had clear MLH1 foci present along the chromosomes (Fig. 5, I and J). However, in *Sycp1^L106E^* and *Sycp1*^*L102E*/*L106E*^ mutants, MLH1 staining was completely absent (Fig. 5, K and L). Together, these studies suggest that meiotic recombination is initiated in these mutants, but there is a failure to form crossovers when N-terminal self- assembly is lost.

### SIX6OS1 and SYCE3 binding are retained in *Sycp1^L106E^* and *Sycp1^L102E/L106E^* mice

**Fig. 6.**
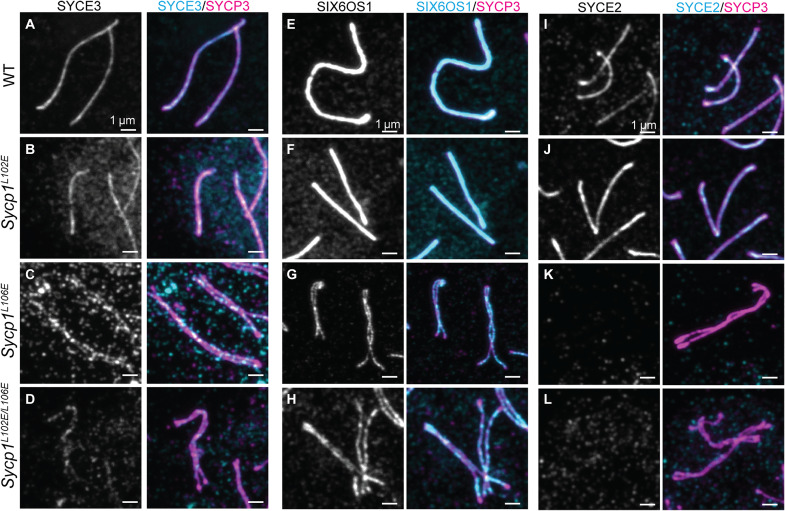
SIX6OS and SYCE3, but not SYCE2, are present in *Sycp1^L106E^* and *Sycp1^L102E/L106E^* mutants. (**A** to **D**) Chromosome spreads stained with SYCE3 (gray/cyan) and SYCP3 (magenta) show reduced SYCE3 staining in *Sycp1^L106E^* and *Sycp1^L102E/L106E^* pachytene-like nuclei. (**E** to **H**) Chromosome spreads stained with SIX6OS (gray/cyan) and SYCP3 (magenta) show SIX6OS staining in *Sycp1^L106E^* and *Sycp1^L102E/L106E^* pachytene-like nuclei. (**I** to **L**) Chromosome spreads stained with SYCE2 (gray/cyan) and SYCP3 (magenta) show no SYCE2 staining in *Sycp1^L106E^* and *Sycp1^L102E/L106E^* pachytene-like nuclei. All images are at the same exposure. Scale bars, 1 μm. Minimum of three animals analyzed per a genotype.

Last, we wanted to determine how the overall SC structure was affected by the loss of head-to-head assembly in these mutants. Work examining mutants of CE proteins have established that SYCE3 interacts with SYCP1 to create a scaffold for SYCE1/SIX6OS and SYCE2/TEX12 localization (*24*). When SYCP1 is absent, the entire central region is lost with no loading of CE proteins SYCE1, SYCE2, and SYCE3. We stained meiotic spreads with antibodies against multiple CE proteins to test what happens to CE protein localization in *Sycp1^L106E^* and *Sycp1*^*L102E*/*L106E*^ mice when mutant SYCP1 is present, but the SC is not synapsed. First, we examined SYCE3 as it is predicted to be important for the loading of the rest of the CE proteins (*19*, *24*). In WT and *Sycp1^L102E^* pachytene nuclei, SYCE3 staining was present on synapsed chromosomes at pachytene. SYCE3 staining was still present along the unsynapsed chromosomes in *Sycp1^L106E^* and *Sycp1^L102E/L106E^* pachytene-like nuclei (Fig. 6, A to D, and fig. S6, A to E). We quantified the intensity of SYCE3 in all genotypes and found that SYCE3 intensity was decreased in *Sycp1^L102E^*, *Sycp1^L106E^*, and *Sycp1^L102E/L106E^* nuclei (fig. S6, A to E). However, this decrease was not significant. This was likely due to variability in the SYCE3 intensity in WT nuclei. We suspect that variability was present in the quantification due to differences in background staining.

Next, we examined SIX6OS1 localization. SIX6OS1 localized to the synapsed region of the chromosomes in WT and *Sycp1^L102E^* nuclei (Fig. 6, E and F, and fig. S6, F and G). However, in *Sycp1^L106E^* and *Sycp1^L102E/L106E^* pachytene-like nuclei, SIX6OS1 localized along the chromosomes in a similar pattern to SYCP3 (Fig. 6, G and H, and fig. S6, H and I). Quantification of SIX6OS1 intensity confirmed that the amount SIX6OS1 present was very similar in all genotypes with only *Sycp1^L106E^* mice exhibiting a slight decrease (fig. S6J; *P* = 0.034). Last, we stained for CE elongation factor SYCE2, which localized to the synapsed region in WT and *Sycp1^L102E^* pachytene nuclei but was completely absent from *Sycp1^L106E^* and *Sycp1^L102E/L106E^* pachytene nuclei (Fig. 6, I to L, and fig. S6, K to O). This absence was confirmed by intensity quantifications (fig. S6O). Therefore, although CE initiation proteins SYCE3 and SIX6OS1 were retained to some extent, SYCE2 was not recruited. This is consistent with the known direct interaction of SYCE3 with the region of SYCP1 downstream of, and independent of, αNtip head-to-head assembly (*24*). Further, it suggests that synapsis is required for recruitment and/or assembly of SYCE2-TEX12 fibers within the CE.

## DISCUSSION

**Fig. 7.**
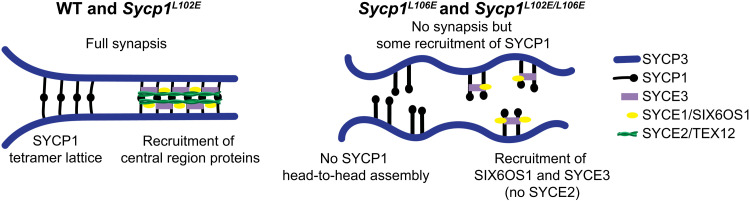
Model of the SC in WT, *Sycp1^L102E^*, *Sycp1^L106E^*, and *Sycp1^L102E/L106E^* mice. In WT and *Sycp1^L102E^*, SYCP1 tetramerizes normally, and all the CE proteins are recruited (SYCE3, SYCE1/SIX6OS1, and SYCE2/TEX12) to fully synapsed chromosomes. In *Sycp1^L106E^* and *Sycp1^L102E/L106E^* mice, SYCP1 localizes to the chromosome axes but does not successfully form head-to-head interactions. Further, SYCE3 and SIX6OS localize to the chromosomes but SYCE2 is unable to localize without full synapsis and SYCP1 head-to-head assembly.

Here, we report that αNtip-mediated head-to-head assembly of SYCP1 is required for chromosome synapsis and meiosis in male mice. In previous work, we demonstrated that human SYCP1 head-to-head assembly is disrupted in vitro by deleting the αNtip or introducing mutations V105E/L109E (*23*). However, the role of head-to-head assembly in SC formation had not previously been tested in vivo. We targeted αNtip head-to-head assembly in mouse meiosis by introducing mutations L102E and L106E, which correspond to human V105E and L109E, respectively. In agreement with in silico MD simulations, L102E had no apparent effect on synapsis, and L106E resulted in complete loss of synapsis, failure of CE assembly, and failure of meiosis in vivo. Thus, we find that αNtip-mediated head-to-head assembly, which is dependent on leucine residue m.L106/h.L109 in SYCP1, is essential for the structure and function of the SC in meiosis. While synapsis was disrupted, the SYCP1 C terminus was still recruited to the meiotic chromosome axis (Fig. 7). Further, we observed disparate consequences on the recruitment of CE proteins (Fig. 7). The sequence that encodes the αNtip and its surrounding αNend head-to-head structure is highly conserved in vertebrates and some plants. Thus, we speculate that αNtip-mediated head-to-head assembly may represent a common mechanism of transverse filament assembly within the SC across kingdoms of life.

### Why does L106E but not L102E disrupt synapsis?

Although αNtip amino acids m.L102/h.V105 and m.L106/h.L109 are conserved as leucine residues in SYCP1 across vertebrates, only the latter residue is conserved in plants. The SYCP1 αNend crystal structure and mouse homology model show that m.L106/h.L109 are located at the core of the SYCP1 αNtip head-to-head interface, whereas m.L102/h.V105 are partially solvent exposed. Hence, the conservation and structural data indicated that glutamate mutation of SYCP1 m.L106/h.L109 is likely to have a stronger role in disrupting the head-to-head interface, whereas glutamate mutation of m.L102/h.V105 may be partly tolerated. Accordingly, MD simulations indicated that the human αNend head-to-head structure was readily disrupted by the L109E mutation, with only a marginal reduction in stability of the double V105E/L109E mutant, whereas the individual V105E mutation had only a very slight destabilizing effect on the head-to-head interface. Therefore, our in silico analysis explains the ability of L106E in SYCP1, but not L102E, to disrupt synapsis in mouse meiosis. Further, its agreement with the mouse phenotypes strongly supports the conclusion that the same αNtip-mediated head-to-head assembly interactions of SYCP1 are responsible for its role in synapsis in vivo.

### How does mutant SYCP1 interact with chromosomes in *Sycp1^L106E^* and *Sycp1^L102E/L106E^* mice?

Our data suggest that replacement of L106 with glutamic acid disrupts the ability of SYCP1 to form head-to-head interactions. In *Sycp1^L106E^* and *Sycp1*^*L102E*/*L106E*^ mice, homologous chromosomes align and SYCP1 colocalizes with SYCP3 on the unsynapsed chromosomes (Fig. 4). SYCP1^L106E^ and SYCP^L102E/L106E^ appear to be capable of localizing to the homologs but not establishing the stable N-terminal interactions necessary for synapsis to occur. The retention of chromosome localization is expected as DNA binding sites at the end of the coiled-coil and in the unstructured C terminus are unaffected by the mutations. On the basis of antibody staining, SYCP1 does not appear to be loaded onto chromosomes at the same levels as in WT. This could be due to many factors. A loss of stable SYCP1 interaction with the axis may be due to a lack of cooperative assembly in the absence of synapsis or a less stable SYCP1 protein may have been degraded. Further, this could be because the signal is split between the two homologs compared to the combined signal that is present when WT chromosomes are synapsed.

### Why are some CE proteins present without head-to-head assembly of SYCP1 but not others?

Previous work supports a model of SC assembly where SYCP1 is loaded on chromosomes first, followed by SYCE3, and then SYCE1-SIX6OS1 and SYCE2-TEX12 (*24*). *Sycp1^L106E^* and *Sycp1^L102E/L106E^* mutants provide a unique opportunity to better understand the role of SYCP1 head-to-head interactions in SC assembly and stabilization as other *Sycp1* mutants do not assemble SYCP1 along the axes (*8*). In *Syce1^−/−^* and *Syce3^−/−^* mice where synapsis does not occur, SYCP1 assembles on individual axes in decreased amounts and fragmented patterns, which may be similar to SYCP1 staining in *Sycp1^L106E^* and *Sycp1^L102E/L106E^* mutants (*19*, *25*). However, in those *Syce1^−/−^* and *Syce3^−/−^* mice, the interactions between other CE proteins are disrupted, making it difficult to examine the relationship between SYCP1 head-to-head assembly and assembly of the central region. Our analysis showed that when head-to-head assembly was lost in *Sycp1^L106E^* and *Sycp1^L102E/L106E^* mice, both SIX6OS1 and SYCE3 were present, but SYCE2 was never visible (Fig. 6). It appears that even without SYCP1 N-terminal interactions, SYCE3 can still interact with SYCP1. This agrees with our biochemical findings that SYCE3 binds to a site downstream of the αNtip and independent of its self-assembly (*24*). The presence of SIX6OS1 suggests that in the absence of full synapsis, SYCE3 can recruit the SYCE1-SIX6OS1 complex but not SYCE2-TEX12. The lack of SYCE2-TEX12 likely represents a requirement for correctly bioriented SYCE3 assemblies to support the cooperative recruitment and fibrous self-assembly of SYCE2-TEX12.

Central region SC proteins are dependent on different types of interactions, including many low-affinity interactions, to establish full synapsis between homologs. The complexity of interactions between SC proteins is likely what gives it the flexibility to perform many functions necessary for meiosis to progress. Uncovering what establishes and supports interactions between SC proteins is key to better understanding meiotic recombination.

## MATERIALS AND METHODS

### Homology modeling

A model of the mouse SYCP1-αN (amino acids 98 to 172) head-to-head tetrameric structure was generated by MODELLER (*34*), using the Structurpedia web server (http://www.farooqed.com/mod/). Homology modeling was performed using the human SYCP1-αN (amino acids 101 to 175) head-to-head tetramer in closed conformation from Protein Data Bank (PDB) accession 6F5X (*23*) as the template, with the alignment provided below (sequence identity = 82%).


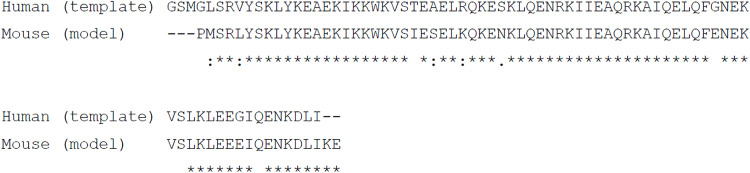


Molecular structure images were generated using the PyMOL Molecular Graphics System, version 2.0.4, Schrödinger LLC.

### Molecular dynamics

MD simulations were performed using AMBER ff19SB and OPC force fields (*35*) in OpenMM (*36*), run locally on NVIDIA GeForce RTX 3090 GPU cards through a Google Colab notebook that was modified from the “Making-it-rain” cloud-based MD notebook (*37*). The human SYCP1-αN (amino acids 101 to 175) head-to-head tetramer in closed conformation was taken from PDB accession 6F5X (*23*), and point mutations V105E and L109E were introduced to all chains using the PyMOL Molecular Graphics System, version 2.0.4, Schrödinger LLC. The WT, V105E, L109E, and V105E/L109E structures and the mouse WT, L102E, L106E, and L102E/L106E modeled structures were placed in water boxes 12 Å larger than the structures and were neutralized at a KCl concentration of 150 mM, by AMBER tleap (*35*). The structures were equilibrated for 200 ps and then run for 100 ns at 310 K and 1-bar pressure, using periodic boundary conditions, with the Langevin middle integrator and Monte Carlo barostat, with integration times of 2 fs. Forces of 0, 0.025, 0.05, 0.075, and 0.10 kJ mol^−1^ nm^−1^ were applied to each atom, directed along the axial *z* axis (*c* axis of the crystal structure), in opposite directions for each constituent dimer of the tetramer. Runs were repeated three times, for each mutant, in each force condition. MD trajectories were analyzed using pytraj (*38*, *39*).

### CRISPR-Cas9 design

CRISPR-Cas9 technology was used to engineer new mouse strains containing mutations in the *Sycp1* gene. Potential guide RNA target sites were designed using CRISPR/Cas9 target online predictor (CCTOP) (*40*). The potential target sites were evaluated using the predicted on-target efficiency score and the off-target potential (*41*). The guide RNA target site was selected near the mutation sites. The sequence was ordered as Alt-R CRISPR-Cas9 crisper RNA (crRNA) from Integrated DNA Technologies (IDT). The crRNA was hybridized with a universal tracrRNA (IDT) to form a full-length guide RNA. Ribonucleoprotein (RNP) was prepared with 6 μM full-length guide RNA and 1.2 μM Cas9 protein (IDT).

The donor templates contained the mutation of interest and blocking mutations to prevent guide RNA reannealing. The nontargeting strand was ordered as an Ultramer (IDT) with three phosphorothioate bonds on each end. For each electroporation, 400 ng of single-stranded oligo donor was used.

### Genotyping

Tissue from resulting animals was lysed using QuickExtract DNA Extraction Solution (Epicentre) to release the genomic DNA. Polymerase chain reaction (PCR) was performed to amplify the specific genomic location, followed by a second round of amplification to incorporate sample-specific dual barcodes. All amplicons were pooled and size-selected using the ProNex Size-Selective Purification System (Promega). Cleaned pools were quantified on a Qubit Fluorometer and then ran on an Agilent Bioanalyzer to check sizing and purity. Purified pools were run on an Illumina MiSeq 2 × 250 flow cell. The resulting sequence data were demultiplexed, and read pairs were joined. On-target indel frequency and expected mutations were analyzed using CRIS.py (*42*). After lines were established, genotyping was completed by Transnetyx using real-time PCR.

### Embryo electroporation

All animal research was approved by the Stowers Institute for Medical Research Institutional Animal Care and Use Committee. Genetically modified mice for this study were generated by one-cell mouse embryo electroporation as previously described (*43*). Briefly, immature C57BL/6 female mice (3 to 4 weeks of age) were superovulated by intraperitoneal administration of 5 IU of pregnant mare serum gonadotrophin followed 46 hours later with 5 IU of human chorionic gonadotropin. Females were then mated with C57BL/6 stud males and checked for the presence of copulatory plugs the following morning as indication of successful mating. Fertilized embryos were collected from the oviducts of the successfully mated females and stored in an incubator at 37°C, 5.0% CO_2_ in KSOM media until electroporation.

One-cell mouse embryo electroporation was performed using the NEPAGENE NEPA21 Type II electroporator (Bulldog-Bio, Portsmouth, NH). Fertilized embryos were washed twice in Opti-MEM I media, and approximately 50 embryos were transferred to glass slide equipped with platinum electrodes forming a chamber with a 5-mm gap (NEPAGENE slide electrode CUY505P5). The embryos were placed in a straight line in the electrode chamber in approximately 45-μl total volume of CRISPR RNP solution. Electroporation conditions were as follows: poring pulse of 225 V (1-ms on, 50-ms interval) × 4; transfer pulse of 20 V (50-ms on, 50-ms interval) × 5. Immediately following electroporation, the embryos were removed from the electrode chamber and washed in M2 media. Embryos were then cultured for several hours in KSOM media in a CO_2_ incubator. Following incubation, approximately 20 viable one-cell electroporated embryos were surgically transferred to the oviduct of 0.5 days post-coitum pseudopregnant recipient CBA × B10 female mice. Pups born from the recipient dams were subsequently weaned at 21 days of age, and a tissue sample was taken for genotyping to confirm the desired mutation. Positive G0 males were backcrossed to C57BL/6 females. A minimum of two back crosses to WT mice were completed before analyzing mutant mouse lines.

### Fertility testing

To examine the fertility and reproductive capabilities of the genetically altered mouse lines, mating trials were performed as a means of fertility testing. Sexually mature homozygous males containing the correct *Sycp1* mutation were paired with sexually mature (8 to 10 weeks of age) female mice of the same background, C57BL/6. Female mice were checked daily for the presence of a copulatory plug as confirmation of successful mating. Plug dates were recorded for each female. The females remained housed with the male mice and were carefully monitored for signs of pregnancy throughout gestation. If the female appeared to be pregnant at 2 weeks following the plug date, she was moved to an individual cage and allowed to carry the litter to term to assess litter size and health status. If plugged females did not appear pregnant, they were allowed to stay in the male’s cage for continued plug checking. All females were removed from the male’s cage following 4 weeks of mating, and a new female was introduced for a second round of mating trials. To test female fertility, the same process was used but with C57BL/6 males and sexually mature homozygous females containing the correct *Sycp1* mutation.

Male mice homozygous for the *Sycp1^L106E^* or *Sycp1^L102E/L106E^* mutation were capable of successfully mating and plugging the WT females; however, no females became pregnant even after being plugged at least twice by the mutant male. This was consistent for two rounds of mating trials demonstrating that the males are capable of mating and plugging but are not fertile and cannot sire offspring.

### Chromosome spreads and antibody staining

Seminiferous tubules were digested from each testis, washed in hypotonic buffer (0.15 M sucrose, 5 mM EDTA, and 0.1 mM phenylmethylsulphonyl fluoride in water) two times and then incubated in hypotonic buffer for 45 min on ice. The supernatant was replaced with 200 μl of hypotonic buffer, and seminiferous tubules were homogenized with pestle. Spermatocyte suspension was mixed with fixative (1.6% paraformaldehyde, 0.2% Triton X-100, 0.05 M sucrose, and 5 mM EDTA in water) and applied on slides. Slides were placed in humidified chamber, fixed for 3 hours, and then air-dried for 30 min at room temperature. Slides were washed with wash buffer (0.1% propylene glycol and 0.04% Triton X-100 in water) for 20 min two times, air-dried at room temperature, and then stored at −70°C. For immunostaining, slides were treated with SuperBlock (37580, Thermo Fisher Scientific) for 1 hour at room temperature, incubated with the following primary antibodies: rabbit anti-SYCP1 C terminus (1:200; Abcam, #ab15090), rabbit anti-SYCP1 N terminus [1:500; (*44*)], mouse anti-SYCP3 (1:500; Abcam, #ab97672), rabbit anti-SIX6OS1 [1:50; (*21*)], guinea pig anti-SYCE2 [1:1000; (*25*)], rabbit anti-SYCE3 [1:300; (*19*)], mouse anti-γH2AX [1:50000; Sigma-Aldrich, 05-636-I], and mouse anti-MLH1 (1:100; BD Biosciences, 550838) in phosphate-buffered saline (PBS) containing 25% SuperBlock overnight at 4°C and then incubated with secondary antibodies (Alexa Fluor 488, Alexa Fluor 568, and Alexa Fluor 647, Invitrogen) in PBS for 1 hour at room temperature. Slides were stained with 4′,6-diamidino-2-phenylindole (DAPI) and washed with PBS containing 0.1% Triton X-100 and PBS. Samples were mounted in ProLong Gold (Invitrogen), and slides were stored at 4°C until observation.

### Hematoxylin and eosin staining

Testis was fixed with 4% paraformaldehyde for 16 hours at 4°C and followed by dehydration, paraffin infiltration, and embedding. Testis was sectioned at 5 μm, and slides were stained with hematoxylin for 3 min and eosin for 1 min after deparaffinization. Then, slides were washed with distilled water and air-dried.

### TUNEL staining

Cell apoptosis was detected using a CF488 TUNEL apoptosis detection kit (30064, Biotium, Hayward, CA, USA) according to the manufacturer’s instruction with some modifications. Briefly, slides were deparaffinized, and microwave antigen retrieval was applied at 95°C for 15 min. Then, sections were permeabilized with proteinase K (20 μg/ml) for 30 min at room temperature and washed with PBS five times. Sections were incubated with TUNEL equilibration buffer for 5 min, incubated with TUNEL reaction mix for 3 hours at 37°C, and then washed with PBS containing 0.1% Triton X-100 and bovine serum albumin (5 mg/ml). Slides were counterstained with DAPI and mounted with ProLong Gold.

### Imaging

Chromosome spread and TUNEL images were acquired with an Orca Flash 4.0 scientific complementary metal-oxide semiconductor 100 fps at full resolution on a Nikon Eclipse Ti2 microscope equipped with a Yokagawa CSU W1 10,000 rpm spinning disk confocal system. The spinning disk confocal is equipped with a quad filter for excitation with 405/488/561/640. Emissions filters used to acquire this image were as follows: far-red: 669 to 741 nm, red: 579 to 631 nm, green fluorescent protein: 507 to 543 nm, and DAPI: 430 to 480 nm. A Nikon Plan Apochromat Lambda long working distance 100× objective was used to acquire the image with 50 to 100 ms exposure times. A Nikon Plan Apochromat 100× oil numerical aperture 1.49 objective was used to acquire the image with 50- to 100-ms exposure times.

Structured illumination microscopy (SIM) images were acquired using Lattice SIM technology on the Elyra 7 microscope (Carl Zeiss AG), and the acquisition configuration and immersion oil optimization were similar to a previous publication (*45*). The acquisition was done using a 63× oil immersion objective lens (Plan Apochromat 63×/1.40 oil), the illumination pattern was set to 15 phases, and the *z*-stack spacing was set at 0.1 μm with a range of 2 to 3 μm. The green and red emission ranges are from 495 to 550 nm and 570 to 620 nm, respectively. SIM raw images were processed using the ZEN software (from Zeiss) with manual adjustments for sharpness in the range of 10 to 11.

### Intensity analysis

For each image, the slice with the maximum intensity was identified, and a projection using the two slices above and two slices below the identified slice was performed on all images. Subsequently, individual cells from projected images were manually marked with freehand regions of interest (ROIs) using ImageJ. In addition, a second ROI, representing the background, was added to the ROI manager outside the cell spreads. Using an ImageJ macro, both the area and mean intensity of each ROI were calculated using ImageJ macro. To obtain the final measurements, we conducted a background subtraction by subtracting the intensity values from the background ROIs. The integrated intensity was then calculated as a product of the intensity and area of the ROIs, excluding the background.

### Distance measurements

SIM data were reconstructed using Zeiss’ SIM2 method to enhance the resolution for distance measurements. During SIM2 processing, the signal input signal-to-noise ratio was set to “high,” with 40 iterations and an output sampling of 3. Gaussian fitter was used for the output imaging.

For WT and L102E samples, we used simple neurite tracer for segmenting individual traces; in the case of L102EL106E and L106E samples, manual line ROIs were drawn to trace individual chromosomes. All traces were straightened using the “Straighten” plug-in in ImageJ, with a pixel size of 80.

After straightening, we selected 10 evenly spaced points crossing each chromosome for distance measurement. A line with a thickness of 3 was drawn perpendicularly to the chromosome to measure the line profiles. To identify the positions of the peaks in the profiles, the “find_peaks” function from the scipy.signal library was used. If the profiles exhibited two peaks, they were fitted with a double Gaussian function to precisely locate the peak positions. The distance between these two peaks was then considered as the distance of the tracks.

### Statistical analysis

An unpaired *t* test was used to compare testis weights. *N* values and exact *P* values can be found in Fig. 3. A Fisher’s exact test was used to compare the number of seminiferous tubules with less than five TUNEL cells to the number of seminiferous tubules with five or more TUNEL cells. *N* values and exact *P* values can be found in table S2. A Mann-Whitney *U* test was used to compare intensity quantifications (figs. S5 and S6).

## References

[1] T. Hassold, P. Hunt, To err (meiotically) is human: The genesis of human aneuploidy. Nat. Rev. Genet. 2, 280–291 (2001). 11283700 10.1038/35066065

[2] A. Geisinger, R. Benavente, Mutations in genes coding for synaptonemal complex proteins and their impact on human fertility. Cytogenet. Genome Res. 150, 77–85 (2016). 27997882 10.1159/000453344

[3] C. Rapino, N. Battista, M. Bari, M. Maccarrone, Endocannabinoids as biomarkers of human reproduction. Hum. Reprod. Update 20, 501–516 (2014). 24516083 10.1093/humupd/dmu004

[4] A. Gudeloglu, S. J. Parekattil, Update in the evaluation of the azoospermic male. Clinics (Sao Paulo) 68, 27–34 (2013). 10.6061/clinics/2013(Sup01)04 PMC3583174 23503952

[5] D. Zickler, N. Kleckner, Recombination, pairing, and synapsis of homologs during meiosis. Cold Spring Harb. Perspect. Biol. 7, a016626 (2015). 25986558 10.1101/cshperspect.a016626 PMC4448610

[6] N. Hunter, Meiotic recombination: The essence of heredity. Cold Spring Harb. Perspect. Biol. 7, a016618 (2015). 26511629 10.1101/cshperspect.a016618 PMC4665078

[7] F. Baudat, Y. Imai, B. de Massy, Meiotic recombination in mammals: Localization and regulation. Nat. Rev. Genet. 14, 794–806 (2013). 24136506 10.1038/nrg3573

[8] F. A. T. de Vries, E. de Boer, M. van den Bosch, W. M. Baarends, M. Ooms, L. Yuan, J.-G. Liu, A. A. van Zeeland, C. Heyting, A. Pastink, MouseSycp1functions in synaptonemal complex assembly, meiotic recombination, and XY body formation. Genes Dev. 19, 1376–1389 (2005). 15937223 10.1101/gad.329705 PMC1142560

[9] M. A. Handel, J. C. Schimenti, Genetics of mammalian meiosis: Regulation, dynamics and impact on fertility. Nat. Rev. Genet. 11, 124–136 (2010). 20051984 10.1038/nrg2723

[10] P. Laissue, Aetiological coding sequence variants in non-syndromic premature ovarian failure: From genetic linkage analysis to next generation sequencing. Mol. Cell. Endocrinol. 411, 243–257 (2015). 25960166 10.1016/j.mce.2015.05.005

[11] T. M. Grishaeva, Y. F. Bogdanov, Conservation and variability of synaptonemal complex proteins in phylogenesis of eukaryotes. International Journal of Evolutionary Biology. 2014, e856230 (2014). 10.1155/2014/856230 PMC4132317 25147749

[12] S. L. Page, R. S. Hawley, The genetics and molecular biology of the synaptonemal complex. Annu. Rev. Cell Dev. Biol. 20, 525–558 (2004). 15473851 10.1146/annurev.cellbio.19.111301.155141

[13] M. Westergaard, D. von Wettstein, The synaptinemal complex. Annu. Rev. Genet. 6, 71–110 (1972). 4269097 10.1146/annurev.ge.06.120172.000443

[14] C. K. Cahoon, R. S. Hawley, Regulating the construction and demolition of the synaptonemal complex. Nat. Struct. Mol. Biol. 23, 369–377 (2016). 27142324 10.1038/nsmb.3208

[15] J. H. Lammers, H. H. Offenberg, M. van Aalderen, A. C. Vink, A. J. Dietrich, C. Heyting, The gene encoding a major component of the lateral elements of synaptonemal complexes of the rat is related to X-linked lymphocyte-regulated genes. Mol. Cell. Biol. 14, 1137–1146 (1994). 8289794 10.1128/mcb.14.2.1137-1146.1994 PMC358469

[16] H. H. Offenberg, J. A. Schalk, R. L. Meuwissen, M. van Aalderen, H. A. Kester, A. J. Dietrich, C. Heyting, SCP2: A major protein component of the axial elements of synaptonemal complexes of the rat. Nucleic Acids Res. 26, 2572–2579 (1998). 9592139 10.1093/nar/26.11.2572 PMC147596

[17] R. L. Meuwissen, H. H. Offenberg, A. J. Dietrich, A. Riesewijk, M. van Iersel, C. Heyting, A coiled-coil related protein specific for synapsed regions of meiotic prophase chromosomes. EMBO J. 11, 5091–5100 (1992). 1464329 10.1002/j.1460-2075.1992.tb05616.x PMC556987

[18] Y. Costa, R. Speed, R. Öllinger, M. Alsheimer, C. A. Semple, P. Gautier, K. Maratou, I. Novak, C. Höög, R. Benavente, H. J. Cooke, Two novel proteins recruited by synaptonemal complex protein 1 (SYCP1) are at the centre of meiosis. J. Cell Sci. 118, 2755–2762 (2005). 15944401 10.1242/jcs.02402

[19] S. Schramm, J. Fraune, R. Naumann, A. Hernandez-Hernandez, C. Höög, H. J. Cooke, M. Alsheimer, R. Benavente, A novel mouse synaptonemal complex protein is essential for loading of central element proteins, recombination, and fertility. PLOS Genet. 7, e1002088 (2011). 21637789 10.1371/journal.pgen.1002088 PMC3102746

[20] G. Hamer, K. Gell, A. Kouznetsova, I. Novak, R. Benavente, C. Höög, Characterization of a novel meiosis-specific protein within the central element of the synaptonemal complex. J. Cell Sci. 119, 4025–4032 (2006). 16968740 10.1242/jcs.03182

[21] L. Gómez-H, N. Felipe-Medina, M. Sánchez-Martín, O. R. Davies, I. Ramos, I. García-Tuñón, D. G. de Rooij, I. Dereli, A. Tóth, J. L. Barbero, R. Benavente, E. Llano, A. M. Pendas, C14ORF39/SIX6OS1 is a constituent of the synaptonemal complex and is essential for mouse fertility. Nat. Commun. 7, 13298 (2016). 27796301 10.1038/ncomms13298 PMC5095591

[22] E. Bolcun-Filas, Y. Costa, R. Speed, M. Taggart, R. Benavente, D. G. De Rooij, H. J. Cooke, SYCE2 is required for synaptonemal complex assembly, double strand break repair, and homologous recombination. J. Cell Biol. 176, 741–747 (2007). 17339376 10.1083/jcb.200610027 PMC2064047

[23] J. M. Dunce, O. M. Dunne, M. Ratcliff, C. Millán, S. Madgwick, I. Usόn, O. R. Davies, Structural basis of meiotic chromosome synapsis through SYCP1 self-assembly. Nat. Struct. Mol. Biol. 25, 557–569 (2018). 29915389 10.1038/s41594-018-0078-9 PMC6606445

[24] J. H. Crichton, J. M. Dunce, O. M. Dunne, L. J. Salmon, P. S. Devenney, J. Lawson, I. R. Adams, O. R. Davies, Structural maturation of SYCP1-mediated meiotic chromosome synapsis by SYCE3. Nat. Struct. Mol. Biol. 30, 188–199 (2023). 36635604 10.1038/s41594-022-00909-1 PMC7614228

[25] E. Bolcun-Filas, R. Speed, M. Taggart, C. Grey, B. de Massy, R. Benavente, H. J. Cooke, Mutation of the mouse Syce1 gene disrupts synapsis and suggests a link between synaptonemal Complex structural components and DNA repair. PLOS Genet. 5, e1000393 (2009). 19247432 10.1371/journal.pgen.1000393 PMC2640461

[26] F. Sánchez-Sáez, L. Gómez-H, O. M. Dunne, C. Gallego-Páramo, N. Felipe-Medina, M. Sánchez-Martín, E. Llano, A. M. Pendas, O. R. Davies, Meiotic chromosome synapsis depends on multivalent SYCE1-SIX6OS1 interactions that are disrupted in cases of human infertility. Sci. Adv. 6, eabb1660 (2020). 32917591 10.1126/sciadv.abb1660 PMC7467691

[27] J. M. Dunce, L. J. Salmon, O. R. Davies, Coiled-coil structure of meiosis protein TEX12 and conformational regulation by its C-terminal tip. Commun Biol. 5, 921 (2022). 36071143 10.1038/s42003-022-03886-9 PMC9452514

[28] L. W. Hemmer, J. P. Blumenstiel, Holding it together: Rapid evolution and positive selection in the synaptonemal complex of *Drosophila*. BMC Evol. Biol. 16, 91 (2016). 27150275 10.1186/s12862-016-0670-8 PMC4857336

[29] J. Fraune, M. Alsheimer, J.-N. Volff, K. Busch, S. Fraune, T. C. G. Bosch, R. Benavente, Hydra meiosis reveals unexpected conservation of structural synaptonemal complex proteins across metazoans. Proc. Natl. Acad. Sci. 109, 16588–16593 (2012). 23012415 10.1073/pnas.1206875109 PMC3478637

[30] Y. Xiang, D. E. Miller, E. J. Ross, A. Sánchez Alvarado, R. S. Hawley, Synaptonemal complex extension from clustered telomeres mediates full-length chromosome pairing in *Schmidtea mediterranea*. Proc. Natl. Acad. Sci. U.S.A. 111, E5159–E5168 (2014). 25404302 10.1073/pnas.1420287111 PMC4260563

[31] G. Hamer, H. Wang, E. Bolcun-Filas, H. J. Cooke, R. Benavente, C. Höög, Progression of meiotic recombination requires structural maturation of the central element of the synaptonemal complex. J. Cell Sci. 121, 2445–2451 (2008). 18611960 10.1242/jcs.033233

[32] S. K. Mahadevaiah, J. M. Turner, F. Baudat, E. P. Rogakou, P. de Boer, J. Blanco-Rodríguez, M. Jasin, S. Keeney, W. M. Bonner, P. S. Burgoyne, Recombinational DNA double-strand breaks in mice precede synapsis. Nat. Genet. 27, 271–276 (2001). 11242108 10.1038/85830

[33] P. B. Moens, N. K. Kolas, M. Tarsounas, E. Marcon, P. E. Cohen, B. Spyropoulos, The time course and chromosomal localization of recombination-related proteins at meiosis in the mouse are compatible with models that can resolve the early DNA-DNA interactions without reciprocal recombination. J. Cell Sci. 115, 1611–1622 (2002). 11950880 10.1242/jcs.115.8.1611

[34] A. Sali, T. L. Blundell, Comparative protein modelling by satisfaction of spatial restraints. J. Mol. Biol. 234, 779–815 (1993). 8254673 10.1006/jmbi.1993.1626

[35] D. A. Case, H. M. Aktulga, K. Belfon, I. Y. Ben-Shalom, J. T. Berryman, S. R. Brozell, D. S. Cerutti, T. E. Cheatham, III, G. A. Cisneros, V. W. D. Cruzeiro, T. A. Darden, R. E. Duke, G. Giambasu, M. K. Gilson, H. Gohlke, A. W. Goetz, R. Harris, S. Izadi, S. A. Izmailov, K. Kasavajhala, M. C. Kaymak, E. King, A. Kovalenko, T. Kurtzman, T. S. Lee, S. LeGrand, P. Li, C. Lin, J. Liu, T. Luchko, R. Luo, M. Machado, V. Man, M. Manathunga, K. M. Merz, Y. Miao, O. Mikhailovskii, G. Monard, H. Nguyen, K. A. O’Hearn, A. Onufriev, F. Pan, S. Pantano, R. Qi, A. Rahnamoun, D. R. Roe, A. Roitberg, C. Sagui, S. Schott-Verdugo, A. Shajan, J. Shen, C. L. Simmerling, N. R. Skrynnikov, J. Smith, J. Swails, R. C. Walker, J. Wang, J. Wang, H. Wei, R. M. Wolf, X. Wu, Y. Xiong, Y. Xue, D. M. York, S. Zhao, P. A. Kollman, *Amber Manual 2022* (2022).

[36] P. Eastman, J. Swails, J. D. Chodera, R. T. McGibbon, Y. Zhao, K. A. Beauchamp, L.-P. Wang, A. C. Simmonett, M. P. Harrigan, C. D. Stern, R. P. Wiewiora, B. R. Brooks, V. S. Pande, OpenMM 7: Rapid development of high performance algorithms for molecular dynamics. PLoS Comput. Biol. 13, e1005659 (2017). 28746339 10.1371/journal.pcbi.1005659 PMC5549999

[37] P. R. Arantes, M. D. Polêto, C. Pedebos, R. Ligabue-Braun, Making it rain: Cloud-based molecular simulations for everyone. J. Chem. Inf. Model. 61, 4852–4856 (2021). 34595915 10.1021/acs.jcim.1c00998

[38] D. R. Roe, T. E. I. Cheatham, PTRAJ and CPPTRAJ: Software for processing and analysis of molecular dynamics trajectory data. J. Chem. Theory Comput. 9, 3084–3095 (2013). 26583988 10.1021/ct400341p

[39] H. Nguyen, D. R. Roe, J. Swails, D. A. Case, *PYTRAJ: Interactive Data Analysis for Molecular Dynamics Simulations*. (2016).

[40] M. Stemmer, T. Thumberger, M. Del Sol Keyer, J. Wittbrodt, J. L. Mateo, CCTop: An intuitive, flexible and reliable CRISPR-Cas9 target prediction tool. PLOS ONE 10, e0124633 (2015). 25909470 10.1371/journal.pone.0124633 PMC4409221

[41] M. Labuhn, F. F. Adams, M. Ng, S. Knoess, A. Schambach, E. M. Charpentier, A. Schwarzer, J. L. Mateo, J.-H. Klusmann, D. Heckl, Refined sgRNA efficacy prediction improves large- and small-scale CRISPR-Cas9 applications. Nucleic Acids Res. 46, 1375–1385 (2018). 29267886 10.1093/nar/gkx1268 PMC5814880

[42] J. P. Connelly, S. M. Pruett-Miller, CRIS.py: A versatile and high-throughput analysis program for CRISPR-based genome editing. Sci. Rep. 9, 4194 (2019). 30862905 10.1038/s41598-019-40896-w PMC6414496

[43] M. Hashimoto, T. Takemoto, Electroporation enables the efficient mRNA delivery into the mouse zygotes and facilitates CRISPR-Cas9–based genome editing. Sci. Rep. 5, 11315 (2015). 26066060 10.1038/srep11315 PMC4463957

[44] K. Winkel, M. Alsheimer, R. Ollinger, R. Benavente, Protein SYCP2 provides a link between transverse filaments and lateral elements of mammalian synaptonemal complexes. Chromosoma 118, 259–267 (2009). 19034475 10.1007/s00412-008-0194-0

[45] C. K. Cahoon, Z. Yu, Y. Wang, F. Guo, J. R. Unruh, B. D. Slaughter, R. S. Hawley, Superresolution expansion microscopy reveals the three-dimensional organization of the *Drosophila* synaptonemal complex. Proc. Natl. Acad. Sci. 114, E6857–E6866 (2017). 28760978 10.1073/pnas.1705623114 PMC5565445

[46] A. M. Waterhouse, J. B. Procter, D. M. A. Martin, M. Clamp, G. J. Barton, Jalview version 2–A multiple sequence alignment editor and analysis workbench. Bioinformatics 25, 1189–1191 (2009). 19151095 10.1093/bioinformatics/btp033 PMC2672624

