# Supplementary material for: SYCP1 head-to-head assembly is required for chromosome synapsis in mouse meiosis
Source: Sci Adv. 2023 Oct 20;9(42):eadi1562. doi: 10.1126/sciadv.adi1562 (PMC10588951; doi:10.1126/sciadv.adi1562)
Supplement: Supplementary file 1 — Figs. S1 to S6 Tables S1 and S2 [file sciadv.adi1562_sm.pdf]

Science Advances
AAAS

Supplementary Materials for

SYCP1 head-to-head assembly is required for chromosome synapsis in mouse meiosis

Katherine Kretovich Billmyre *et al.*

Corresponding author: Owen Richard Davies, owen.davies@ed.ac.uk; R. Scott Hawley, rsh@stowers.org

Sci. Adv. **9**, eadi1562 (2023)
DOI: 10.1126/sciadv.adi1562

This PDF file includes:

Figs. S1 to S6
Tables S1 and S2

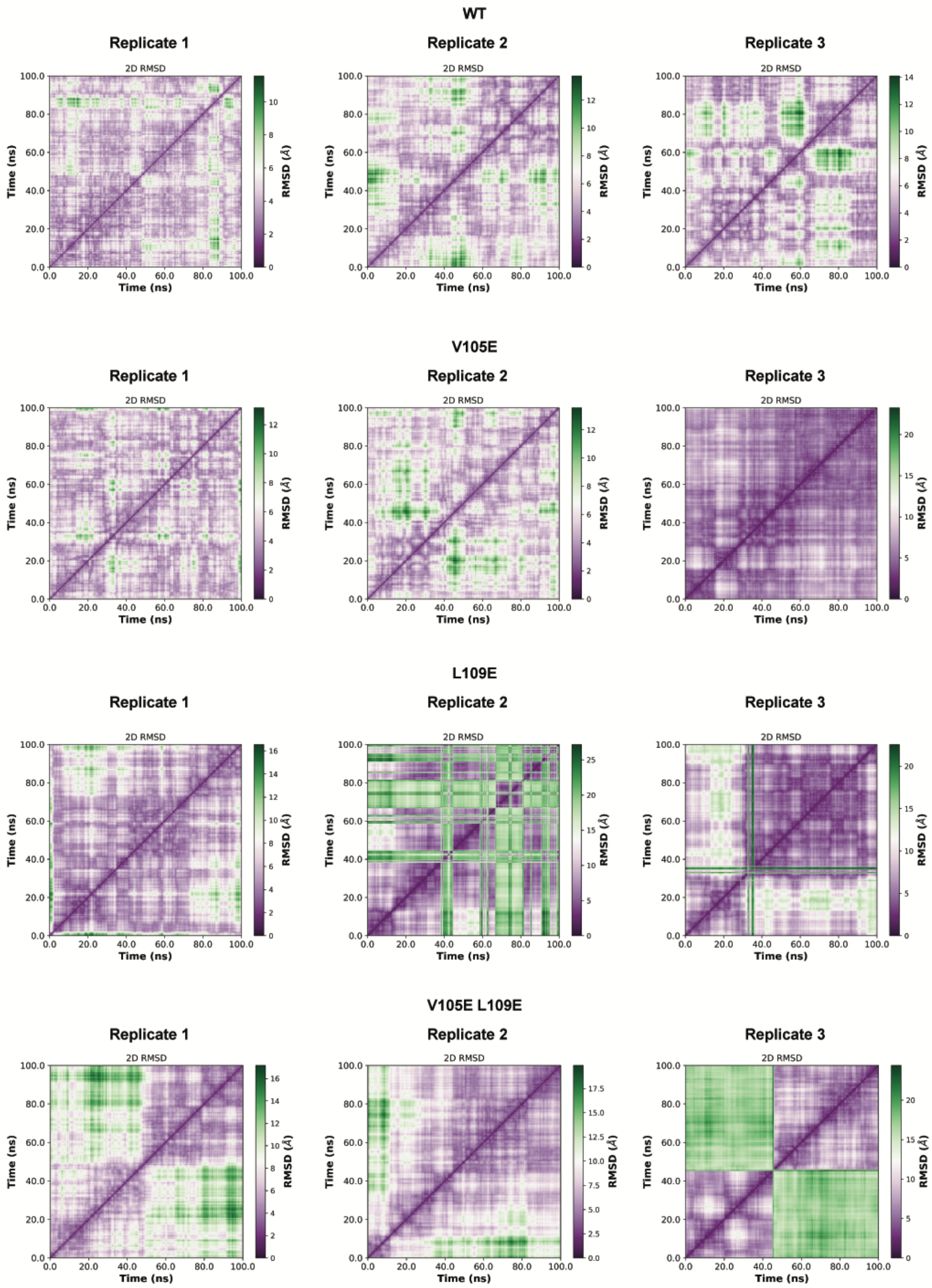

Fig. S1. Molecular dynamics simulations of SYCP1 αN-end

2D r.m.s. deviation plots for the three replicates of 100-ns molecular dynamics simulations of SYCP1 aN-end wild-type (WT), V105E, L109E and V105E/L109E shown in Figure 2A,B.

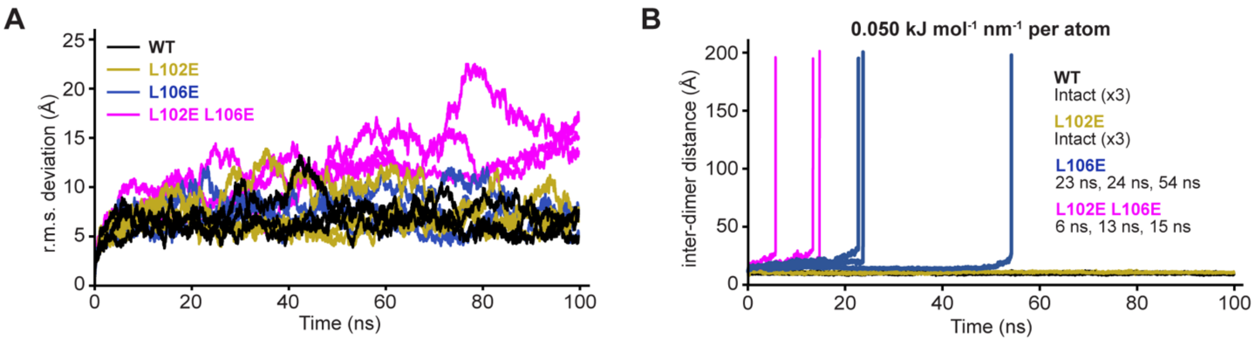

Fig. S2. Molecular dynamics simulations of mouse SYCP1 αN-end

(**A-B**) Molecular dynamics simulations of mouse αNend wildtype (WT), L102E, L106E and L102E/L106E over 100-ns trajectories, in explicit solvent at 37°C (n=3). (**A**) Overall r.m.s. deviations for three replicates after 100-ns simulations. (**B**) Inter-dimer distances plotted for each replicate of SYCP1 αNend WT, L102E, L106E and L102E/L106E steered molecular dynamics, in which forces of 0.050 kJ mol^{-1} nm^{-1} were applied to each atom, directed along the axial axis, in opposite directions for the two constituent dimers. Interface disruption is marked by a sudden increase in inter-dimer distance, and times of disruption are indicated for all replicates.

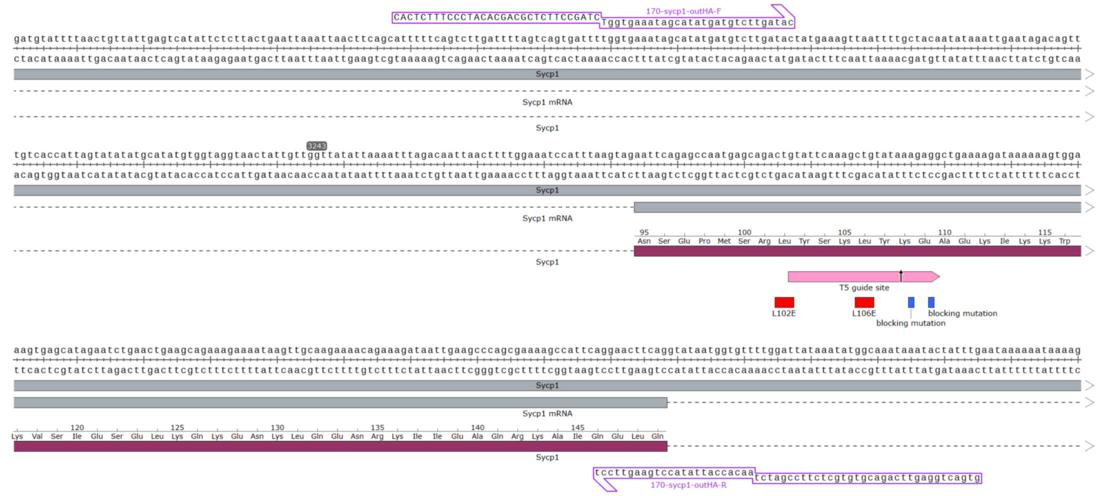

guideRNA (PAM in bold)
Sycp-1_T5: GTATTCAAAGCTGTATAAAG**AGG**

Single-stranded oligo donors
Bold= installed mutation
underlined= silent, blocking mutation
*=phosphorothioate bond

L102E:
c*a*a*ttaacttttggaaatccatttaagtagaattcagagccaatgagcaga**GA**gtattcaaagctgtataaGgaAgctgaaaagataa aaaagtggaaagtgagcatagaatctgaa*c*t*g

L106E:
c*c*a*tttaagtagaattcagagccaatgagcagactgtattcaaag**GA**gtataaGgaAgctgaaaagataaaaaagtggaaagtga gcatagaatctgaa*c*t*g

L102EL106E:
c*a*a*ttaacttttggaaatccatttaagtagaattcagagccaatgagcaga**GA**gtattcaaag**GA**gtataaGgaAgctgaaaagat aaaaaagtggaaagtgagcatagaatctgaa*c*t*g

L102LL106L:
c*a*a*ttaacttttggaaatccatttaagtagaattcagagccaatgagcagact**C**tattcaaagct**C**tataaGgaAgctgaaaagataa aaaagtggaaagtgagcatagaatctgaa*c*t*g

Amplification primers (overhangs for amplicon barcoding underlined)
170-sycp1-outHA-F:
CACTCTTTCCCTACACGACGCTCTTCCGATCTggtgaaatagcatatgatgtcttgatac
170-sycp1-outHA-F:
GTGACTGGAGTTCAGACGTGTGCTCTTCCGATCTaacaccattatacctgaagttcct

Fig. S3. Summary of CRISPR guides and repair templates

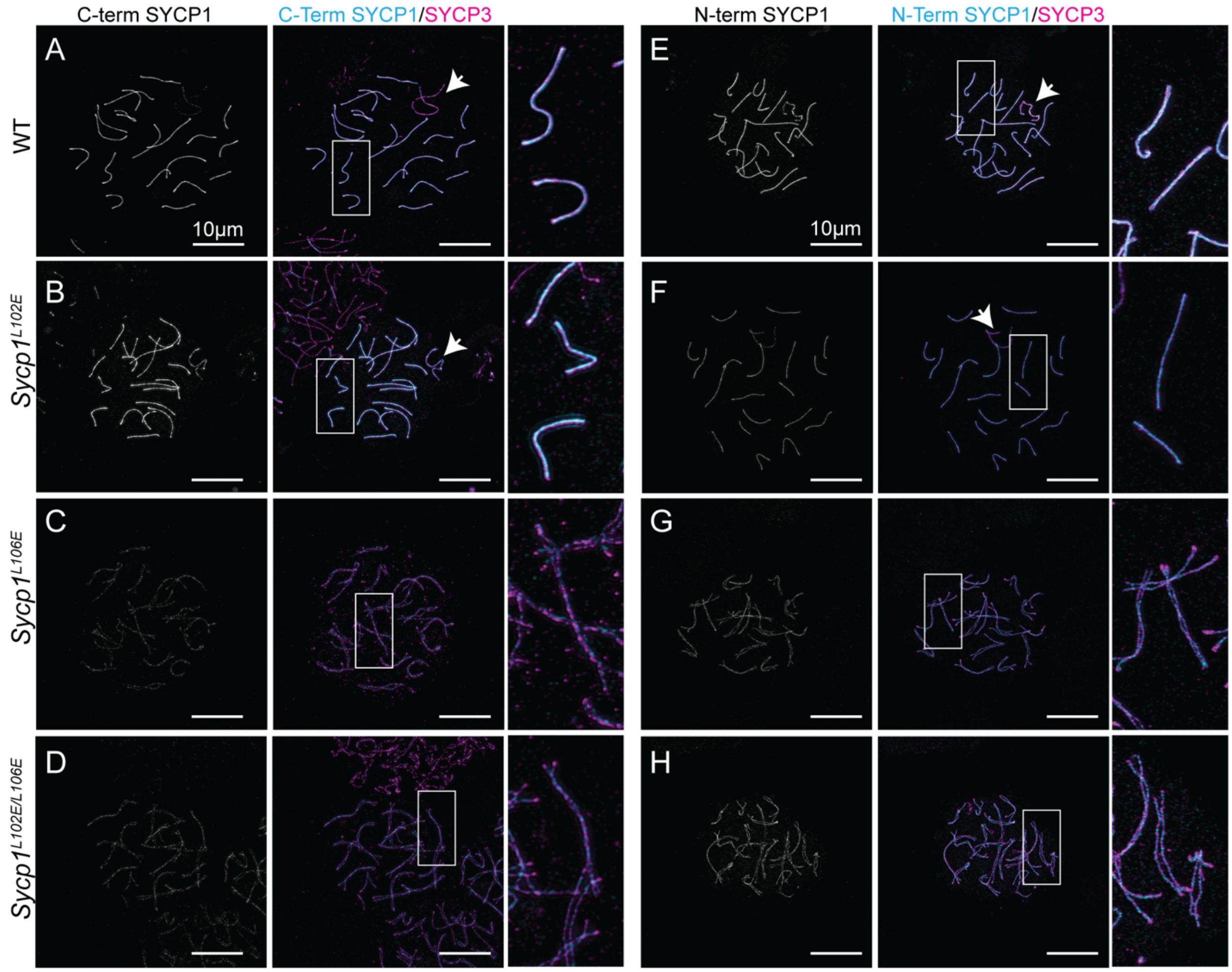

Fig. S4. SYCP1 C-terminal and N-terminal staining present on the chromosome axes in pachytene-like nuclei in Sycp1^{L106E} and Sycp1$^{L102E/L106E}$ mutants.

Pachytene/pachytene-like nuclei stained with antibodies against C-terminal SYCP1(**A-D**) or N-terminal SYCP1 (**E-H**) and SYCP3 and images using structured illumination microscopy in (**A**) WT, (**B**) *Sycp1*^{L102E}, (**C**) *Sycp1*^{L106E}, (**D**) *Sycp1*$^{L102E/L106E}$. The first panel is SYCP1 (grey) and the second is a merge of SYCP1 (cyan) and SYCP3 (magenta) staining. All images are at the same exposure. X and Y chromosomes marked with arrowheads. Scale bar is 10 microns, magnified panel is 3.2x and is the same as main text Figure 4. Minimum of 3 animals analyzed per a genotype.

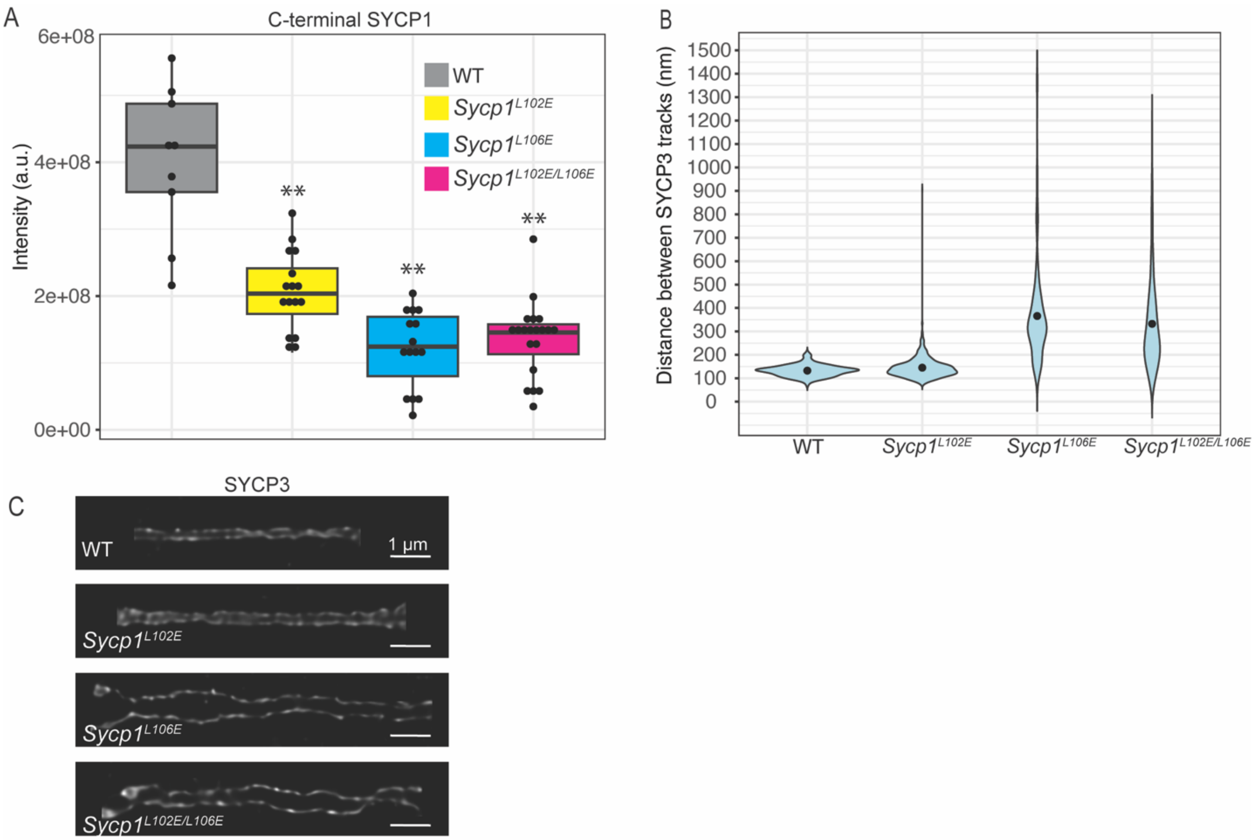

Fig. S5. Quantification of SYCP1 staining and SC width.

(**A**) Quantification of SYCP1 C-terminal intensity in pachytene/pachytene-like nuclei for all genotypes. (**B**) Quantification of the distance between SYCP3 tracks on straightened chromosomes from structured illumination microscopy. (**C**) Examples of straightened chromosomes from WT, *Sycp1^{L102E}*, *Sycp1^{L106E}*, *Sycp1$^{L102E/L106E}$* mice.

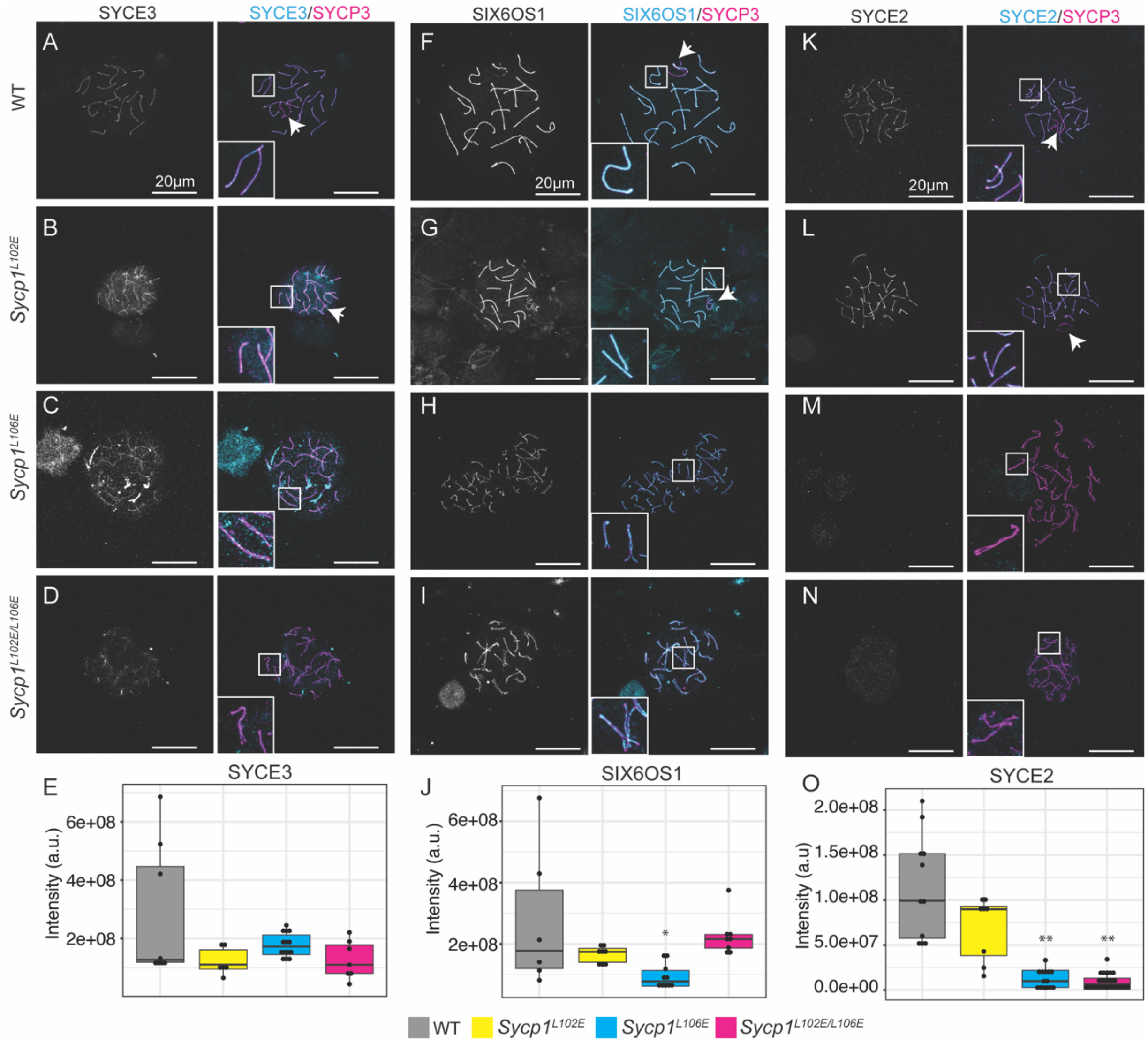

Fig. S6: Quantification of SYCE3, SIX6OS1, SYCE2 staining.

(**A-D**) Chromosome spreads stained with SYCE3 (grey/cyan) and SYCP3 (magenta) show reduced SYCE3 staining in *Sycp1*^{L106E} and *Sycp1*$^{L102E/L106E}$ pachytene-like nuclei. (**F-I**) Chromosome spreads stained with SIX6OS (grey/cyan) and SYCP3 (magenta) show reduced SIX6OS staining in *Sycp1*^{L106E} and *Sycp1*$^{L102E/L106E}$ pachytene-like nuclei. (**K-N**) Chromosome spreads stained with SYCE2 (grey/cyan) and SYCP3 (magenta) show no SYCE2 staining in *Sycp1*^{L106E} and *Sycp1*$^{L102E/L106E}$ pachytene-like nuclei. All images are at the same exposure. Scale bar is 20 microns. Insets are the same magnified images present in main text figure 6. Minimum of 3 animals analyzed per a genotype. (**E, J, O**) Quantification of SYCE3, SIX6OS1, and SYCE2 in pachytene/pachytene-like nuclei from WT, *Sycp1*^{L102E}, *Sycp1*^{L106E}, and *Sycp1*$^{L102E/L106E}$ mice.

**Genotype**	**Phenotype**	**N Value**
C57BL/6J males	100% fertile	8
Sycp1^{L102E} males	100% fertile	3
Sycp1^{L106E} males	100% sterile	6
Sycp1$^{L102E/L106E}$ males	100% sterile	4
C57BL/6J females	100% fertile	12
Sycp1^{L102E} females	100% fertile	5
Sycp1^{L106E} females	100% sterile	4
Sycp1$^{L102E/L106E}$ females	100% sterile	4

Table S1. Summary of fertility assay results

Genotype	% Tubules with 5+ TUNEL positive cells	N Value (Tubules)	N Value (Mice)
C57BL/6J males	1.53%	131	3
Sycp1^{L102E} males	2.25%	89	3
Sycp1^{L106E} males	16.06% ($p<0.0001$)	137	3
Sycp1$^{L102E/L106E}$ males	11.81% ($p=0.0008$)	127	3

Table S2. Quantification of TUNEL positive cells

Tubules were scored as having less than 5 TUNEL cells or 5 or more TUNEL cells. 3 animals analyzed per a genotype. A Fishers exact test was used to test significance on the raw counts.
